# FuNGI: Fungal Nucleolar Genomic Inventory—a comprehensive database of fungal proteins with predicted nucleolar localization signals

**DOI:** 10.1093/database/baag023

**Published:** 2026-05-19

**Authors:** Gnanendra Shanmugam, Chaewon Kim, Surajit De Mandal, Minji Kim, Song Hee Lee, Jaeyoung Choi, Junhyun Jeon

**Affiliations:** Department of Biotechnology, College of Life and Applied Sciences, Yeungnam University, Gyeongsan, Gyeongbuk 38541, Republic of Korea; Department of Convergent Biotechnology and Advanced Materials Science, College of Life Sciences, Kyung Hee University, Yongin 17104, Republic of Korea; Department of Biotechnology, College of Life and Applied Sciences, Yeungnam University, Gyeongsan, Gyeongbuk 38541, Republic of Korea; Department of Biotechnology, College of Life and Applied Sciences, Yeungnam University, Gyeongsan, Gyeongbuk 38541, Republic of Korea; Department of Biotechnology, College of Life and Applied Sciences, Yeungnam University, Gyeongsan, Gyeongbuk 38541, Republic of Korea; Department of Convergent Biotechnology and Advanced Materials Science, College of Life Sciences, Kyung Hee University, Yongin 17104, Republic of Korea; BK21 Interdisciplinary Program in IT-Bio Convergence System, Kyung Hee University, Yongin 17104, Republic of Korea; Department of Biotechnology, College of Life and Applied Sciences, Yeungnam University, Gyeongsan, Gyeongbuk 38541, Republic of Korea; Phytobiome Engineering Research Laboratory, Yeungnam University, Gyeongsan, Gyeongbuk 38541, Republic of Korea

## Abstract

The nucleolus is a well-characterized sub-nuclear compartment primarily responsible for ribosomal RNA (rRNA) synthesis and ribosome biogenesis. In addition to these canonical functions, it plays roles in a variety of other cellular processes. Despite its importance, the contributions of the nucleolus to fungal development and pathogenicity remain poorly understood, especially in filamentous fungi. The structure and function of the nucleolus are regulated by numerous proteins that either reside within it or shuttle between the nucleolus and nucleoplasm, often guided by short amino acid motifs known as nucleolar localization signals (NoLSs). However, comprehensive resources cataloguing nucleolar proteins and their localization signals in fungi are currently lacking, hindering systematic investigations of nucleolar function across species. To address this gap, we developed FuNGI (Fungal Nucleolar Genomic Inventory), a web-based interactive database for the exploration of fungal proteins containing predicted nucleolar localization signals. The current version of FuNGI includes proteins containing predicted nucleolar localization signals and their associated NoLSs across 769 fungal proteomes spanning eight phyla. The database offers a user-friendly interface that enables browsing, retrieval, and comparison of NoLS-containing proteins across multiple species. Each entry integrates sequence-based predictions and functional annotations to support comparative and functional analyses. To our knowledge, FuNGI is the first comprehensive and interactive database dedicated to fungal proteins containing predicted nucleolar localization signals. By enabling systematic and cross-species analyses, FuNGI provides a valuable resource for advancing our understanding of fungal nucleoli and their roles in fungal biology and pathogenicity.

## Introduction

The nucleolus is a specialized sub-nuclear domain primarily responsible for ribosomal RNA (rRNA) synthesis and ribosome biogenesis. In mammals, the nucleolus comprises three distinct structural components: fibrillar centres (FCs), the dense fibrillar component (DFC), and the granular component (GC), which reflect the sequential steps of ribosome production [1]. Over the past two decades, studies have revealed that the nucleolus plays additional roles beyond ribosome assembly. These include functions in DNA damage repair, signal recognition particle assembly, cell cycle regulation, tumour suppression, and oncogenic processes [2–6]. Many of these functions are mediated by the dynamic trafficking of proteins between the nucleoplasm, cytoplasm, and nucleolus [7, 8]. Consequently, the nucleolus must accommodate a high degree of cellular traffic, which necessitates tightly regulated protein-targeting mechanisms.

Although nucleolar localization signals (NoLSs) are not responsible for nuclear entry itself, which is typically mediated by nuclear localization signals (NLSs), they are essential for the selective accumulation of proteins within the nucleolus [9, 10]. NoLSs generally consist of short, basic peptide motifs enriched in lysine (K) and arginine (R) residues. These positively charged sequences promote electrostatic interactions with nucleolar components, including ribosomal RNA and nucleolar proteins, thereby facilitating the retention and proper localization of proteins within the nucleolus [11]. The identification of NoLSs is complicated by their structural diversity and the absence of a conserved consensus motif. These sequences may consist of a single cluster or multiple clusters of basic residues, and their sequence context can vary widely. In addition, the considerable overlap between NoLS and NLS motifs often makes it difficult to clearly distinguish between the two, which presents a challenge for accurate characterization [12].

To address these challenges, computational approaches, including machine learning, have been developed. However, the NoD server remains the sole available resource for NoLS prediction, employing artificial neural networks (ANNs) to identify NoLS regions from protein sequences [13]. Although alternative mechanisms for nucleolar localization likely exist, accumulating evidence suggests that NoLS-mediated targeting is a predominant pathway [14–16]. Consequently, identification of NoLSs provides a practical approach for predicting proteins that may localize to the nucleolus in the proteomes of various organisms. Nucleolar proteomes of higher eukaryotes have been extensively characterized, and several databases exist that compile experimentally verified nucleolar proteins [17]. In contrast, nucleolar proteins in fungi and other lower eukaryotes remain understudied [18, 19], despite the potential to gain evolutionary and functional insights into fungal development and pathogenicity.

Most of the limited knowledge about proteins associated with the nucleolus in fungi has been derived from *Saccharomyces cerevisiae*, but this organism may not fully represent the nucleolar diversity observed across the fungal kingdom [18]. For instance, while *S. cerevisiae* has a simplified bipartite nucleolus, *Schizosaccharomyces pombe* exhibits a tripartite structure [18]. Notably, the filamentous fungi with complex developmental and pathogenic programs are expected to differ even more substantially. However, little is known about the nucleolus in filamentous fungi to date [18]. This highlights the need for a systematic and large-scale effort to explore proteins associated with the nucleolus across diverse fungal phyla, offering insights into the evolution of nucleolar architecture.

To address this gap in knowledge and available resources, we developed FuNGI, a user-friendly and web-based database of fungal proteins containing predicted nucleolar localization signals (NoLSs). We systematically predicted NoLS-containing proteins from 769 fungal proteomes representing eight phyla, using the NoD server. To help users prioritize candidate proteins for further analysis, we incorporated additional computational predictions, including the presence of NoLSs, nuclear localization signals (NLSs), and subcellular localization. Based on these features, the predicted proteins were classified into three confidence levels: high, medium, and low.

## Materials and methods

### Data collection

Fungal protein sequences were retrieved from the UniProt Proteome database [20]. This database was selected because it offers comprehensive, nonredundant, and reference-quality proteomes, and it provides direct links between protein sequences and their corresponding genome assemblies, ensuring data consistency and traceability. Protein annotation in FuNGI was primarily based on the presence of nucleolar localization signals (NoLSs), nuclear localization signals (NLSs), and subcellular localization predictions. These annotations were generated by executing NoD [13], NLStradamus [21], and WoLF PSORT [22] command-line tools on local machines. Bash scripts were used to automate the large-scale processing of all 769 fungal proteomes.

### Data curation pipeline

The fungal proteomes downloaded from the UniProt database were annotated using a multi-step prediction and filtering pipeline. In all three steps of the pipeline, NoLS, NLS, and subcellular localization prediction, the complete proteome of each fungal species was used as input. However, only proteins predicted to contain NoLSs were retained in the final database, and each step of the pipeline is described in detail below:

- First, the full proteome of each species was submitted to the command-line version of the NoD server [13]. Proteins predicted to contain NoLSs (hereafter referred to as *NoLS-positive proteins*) were marked for further analysis. The NoD server, currently the only tool specifically developed for NoLS prediction, utilizes an ANN that processes overlapping windows of 13 amino acid residues across the sequence. A NoLS is assigned when the average output score from the ANN for eight consecutive windows is equal to or greater than 0.8.
- Next, the entire proteome was analysed using NLStradamus [21] to predict nuclear localization signals (NLSs). Each protein was evaluated for the presence and number of NLS segments. While NLS predictions were made for the complete proteome, only the NLS results corresponding to NoLS-positive proteins were retained for inclusion in the final database. NLStradamus was selected based on its superior performance relative to other available tools, such as cNLS Mapper [23], NLSdb [24], and SeqNLS [25] (Supplementary Table S1).
- Subcellular localization prediction was performed on the complete proteome of each fungal species using WoLF PSORT [22]. Although all proteins were analysed, only the localization scores corresponding to NoLS-positive proteins were retained. These scores were used for integration into the FuNGI database and served as inputs for the confidence scoring system. This pipeline ensures that all proteins in each fungal proteome were comprehensively evaluated using three independent prediction tools. By filtering downstream results to include only NoLS-positive proteins, the database maintains a focused and functionally relevant set of entries. This design enables consistent classification of proteins containing predicted NoLSs while preserving the necessary information for confidence assessment.

### Confidence level assignment

To categorize the NoLS-positive proteins by their likelihood of nucleolar localization, we defined three confidence levels: high, medium, and low, based on the combined prediction results from NoD, NLStradamus, and WoLF PSORT. To establish these classification thresholds, we first examined prediction patterns of experimentally validated nucleolar proteins from human (43 proteins) [12] and yeast (140 proteins) [26] (Supplementary Tables S2 and S3). Based on their predicted features, these proteins were grouped into three reference categories: (i) *highly probable nucleolar candidates*, for proteins containing both a NoLS and an NLS and predicted to localize to the nucleus; (ii) *probable*, for proteins containing a NoLS but lacking an NLS and predicted to localize to the cytosol; and (iii) *possible*, for proteins predicted to localize to compartments other than the nucleus or cytosol, regardless of NLS presence.

**Figure 1:**
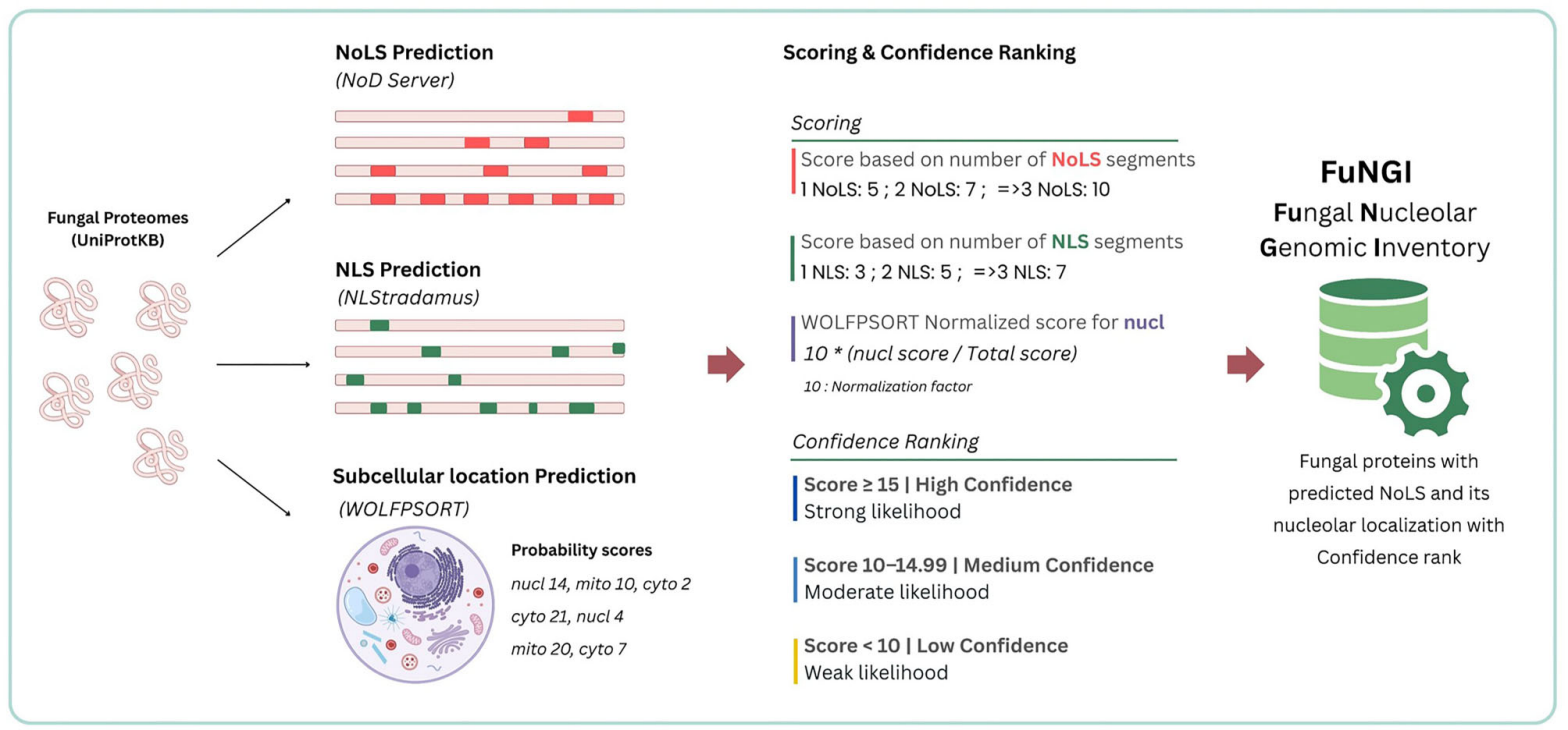
Schematic of the pipeline used in the construction of the FuNGI database. Fungal proteomes from UniProtKB were analysed to predict nucleolar localization signals (NoLSs) using the NoD server, nuclear localization signals (NLSs) using NLStradamus, and subcellular localization using WoLF PSORT. A composite scoring system was applied based on the number of predicted NoLS and NLS segments, along with the normalized WoLF PSORT score for nucleolar localization. Predicted proteins were then categorized into confidence levels (high, medium, or low) based on the final score.

Using these empirically derived categories as a framework, we developed a point-based scoring system to classify the predicted NoLS-containing fungal proteins. This scoring system integrates the number of predicted NoLS and NLS segments, along with a normalized nuclear localization score derived from the WoLF PSORT output. The final confidence score determines the assignment of each protein into one of three categories: high confidence (score ≥15), medium confidence (score ≥10 and <15), and low confidence (score <10) (Fig. 1). This integrative approach ensures that our classification strategy is not solely model-based but also grounded in experimentally supported nucleolar protein profiles.

### Scoring system for confidence assignment

To quantitatively evaluate the nucleolar localization potential of each protein, we developed a scoring system that integrates three components: the number of predicted NoLSs, the number of predicted NLSs, and a normalized nuclear localization score from WoLF PSORT. For NoLS predictions, proteins received 5 points for one NoLS, 7 points for two NoLSs, and 10 points for three or more. For NLSs, proteins were assigned 0 points if no NLS was predicted, 3 points for one NLS, 5 points for two, and 7 points for three or more. To prevent overrepresentation of subcellular localization predictions in the final score, we normalized the raw WoLF PSORT nuclear localization output. If a ‘nucleus’ (*nucl*) score was present, it was divided by the sum of all subcellular localization scores for that protein. The resulting value was then multiplied by 10 to yield the normalized nuclear localization score. If no *nucl* score was present, the nuclear contribution was set to zero.

For example, if the PSORT output for a protein included nucleus = 16.5, mitochondrion-nucleus = 11.0, cytosol = 6.0, and mitochondrion = 4.5, the total score would be 38.0, and the normalized nuclear localization score would be (16.5 ÷ 38.0) × 10 ≈ 4.34. In contrast, if the ‘nucleus’ category was absent (e.g. only mitochondrion-nucleus = 11.0, cytosol = 6.0, and mitochondrion = 4.5), the total would be 21.5, and the normalized nuclear score would be 0.

The final confidence score for each protein was calculated by summing the NoLS score, the NLS score, and the normalized nuclear localization score. Based on this composite score, each protein was classified into a high, medium, or low confidence category as defined above. This integrated scoring strategy ensures consistent and biologically meaningful classification of proteins containing predicted nucleolar localization signals across diverse fungal species.

### Database implementation

FuNGI is hosted on an Apache web server running in a Linux environment. The interactive user interface was developed using Bootstrap version 5.3, with Highcharts, jQuery, and standard JavaScript libraries supporting dynamic charting and interactive data tables. Query processing and data retrieval are managed using PHP version 7.4, which interfaces with a MariaDB (v5.5.68). This database stores comprehensive information, including proteome and protein entries, NoLS and NLS predictions, WoLF PSORT subcellular localization results, and functional annotations, all linked to NCBI Taxonomy identifiers. The NGL Viewer [27] is integrated to display three-dimensional AlphaFold-predicted protein structures obtained from the AlphaFold Protein Structure Database [28], with predicted NoLS segments highlighted for visualization. The current version of FuNGI is compatible with all major modern web browsers, including Google Chrome, Mozilla Firefox, Safari, Opera, and Microsoft Edge.

## Results and discussion

### Database content

**Figure 2:**
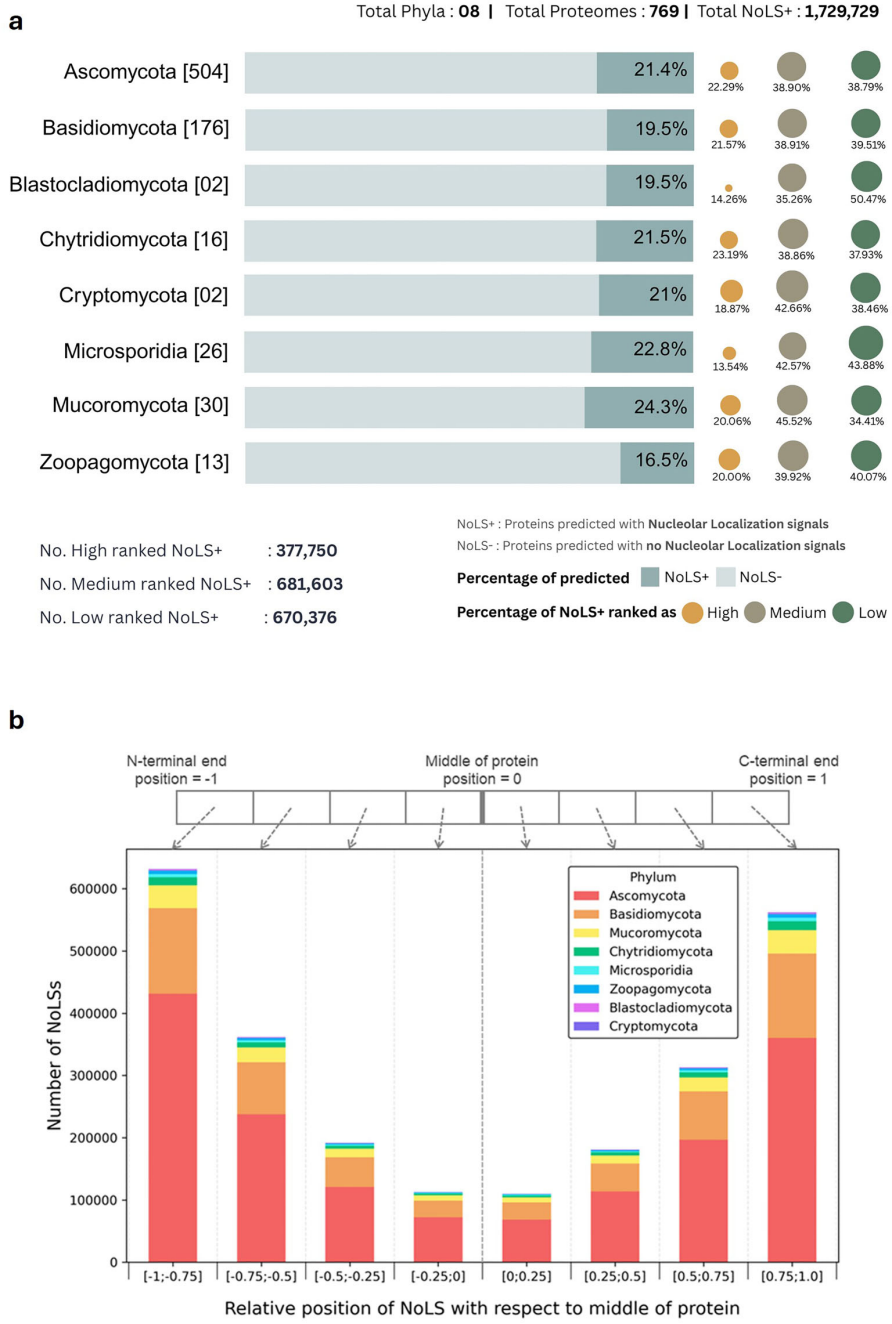
Overview of nucleolar localization predictions. (a) Summary statistics of the FuNGI database. The total number of proteomes analysed from each fungal phylum is indicated in square brackets. Horizontal bars represent the proportion of proteins predicted to contain nucleolar localization signals (NoLS+) versus those without (NoLS−). The accompanying dots, plotted to the right of each bar, denote the distribution of NoLS-containing protein predictions across three confidence ranks — high, medium, and low — as indicated in the legend. The lower panel summarizes the total number of NoLS+ predictions categorized by confidence rank in the current version of FuNGI. (b) Positional distribution of predicted NoLS segments in full-length proteins. The relative positions of NoLSs were normalized to the midpoint of full sequence length, with each stacked bar subdivided by fungal phylum, as indicated in the legend. NoLSs are predominantly localized near the N-terminal and C-terminal ends of proteins.

The current version of FuNGI contains 769 fungal proteomes representing eight fungal phyla. The database includes detailed statistics, such as the number of proteomes per phylum, the total number of proteins analysed, the number of proteins predicted to contain NoLSs, and the distribution of these proteins across the defined confidence categories (high, medium, and low). A summary of these statistics is presented in Fig. 2a.

Analysis of these data reveals that the proportion of proteins predicted to contain NoLS motifs varies substantially across fungal species. For example, while less than 2% of proteins in *Aspergillus nidulans* were predicted to contain NoLS motifs, ~30% of proteins in *Chaetomium globosum* showed predicted NoLS signals. Such variation may reflect differences in proteome composition among species, including variation in amino acid composition and the prevalence of basic residue-rich regions that can influence NoLS prediction. In addition, biases inherent to sequence-based motif prediction tools, as well as differences in genome annotation quality and proteome completeness, may influence prediction outcomes.

Beyond species-level variation, analysis of the positional distribution of predicted NoLSs within full-length proteins shows that NoLSs predominantly occur near the protein termini (Fig. 2b). This positional bias suggests that NoLSs tend to reside in accessible regions of the protein.

These observations highlight that NoLS predictions should be interpreted as indicators of potential nucleolar targeting rather than definitive evidence of nucleolar localization, and the database should therefore be regarded as a resource of candidate proteins containing predicted NoLS motifs.

### Database applications

#### Frontend interface

**Figure 3:**
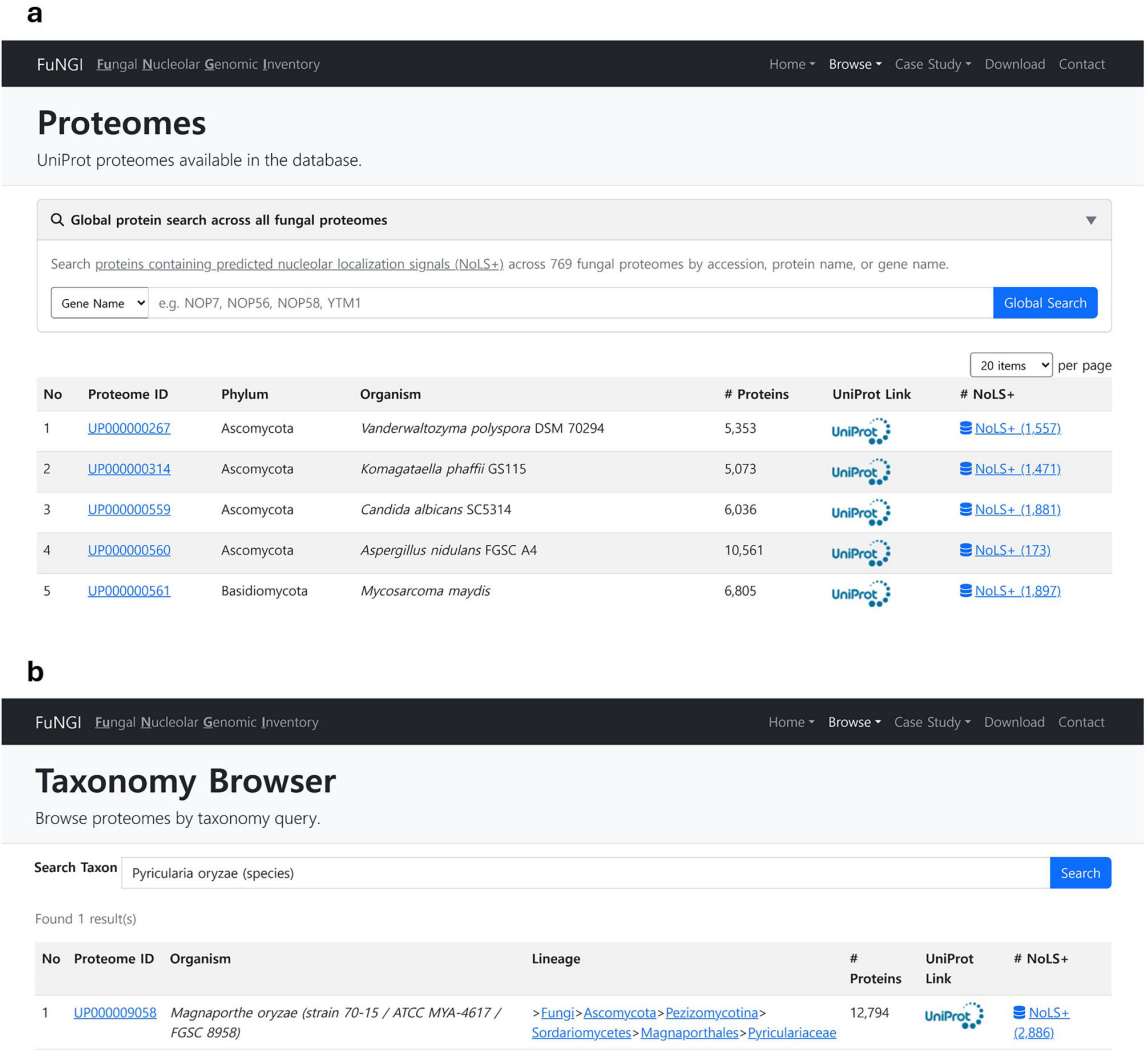
Snapshot of user interface for exploring proteomes in FuNGI. (a) The Proteomes page lists UniProt proteomes available in the database, allowing users to control the number of entries displayed per page and search for specific species. (b) The Taxonomy Browser enables users to search fungal proteomes by genus or species name using a keyword-based query. Both interfaces display organism name, phylum, total number of proteins, UniProt link, and the number of proteins predicted to contain nucleolar localization signals (NoLSs).

FuNGI was developed as a specialized resource to support researchers in the fungal community in exploring and analysing proteins containing predicted nucleolar-localization signals. The platform offers an intuitive web interface for browsing NoLS-predicted proteins across a wide range of fungal species. Two primary navigation options are available: the Proteome Browser and the Taxonomy Browser. To facilitate species selection, the Proteome Browser presents an interactive, paginated table listing all available proteomes in the database. Users can control the number of entries displayed per page and search within the list by species or proteome ID (Fig. 3a). Alternatively, the Taxonomy Browser allows users to navigate across hierarchical taxonomic levels, from phylum to genus or species, via an autocomplete keyword search field (Fig. 3b). Both search methods return a results table that lists fungal species along with organism name, phylum, total number of proteins, and the number of proteins predicted to contain nucleolar localization signals (NoLSs). Within this table, clicking the Proteome ID directs users to a species-specific summary page containing detailed statistics on the NoLS-containing proteins of the selected organism.

Additionally, by clicking the NoLS-positive (NoLS+) proteins link, users can view a complete list of predicted proteins from the selected species. This table includes key information such as a unique identifier (FuNGI_ID), gene name, protein name, UniProt accession number, the number of predicted NoLS and NLS segments, the predicted dominant subcellular compartment, and the associated nucleolar localization confidence rank. In addition, FuNGI provides a global search option that allows users to query a protein name, gene name, or accession across all fungal proteomes rather than restricting searches to a single species. This function facilitates cross-species exploration of conserved nucleolar proteins and supports broader comparative analyses.

### Individual proteome information

**Figure 4:**
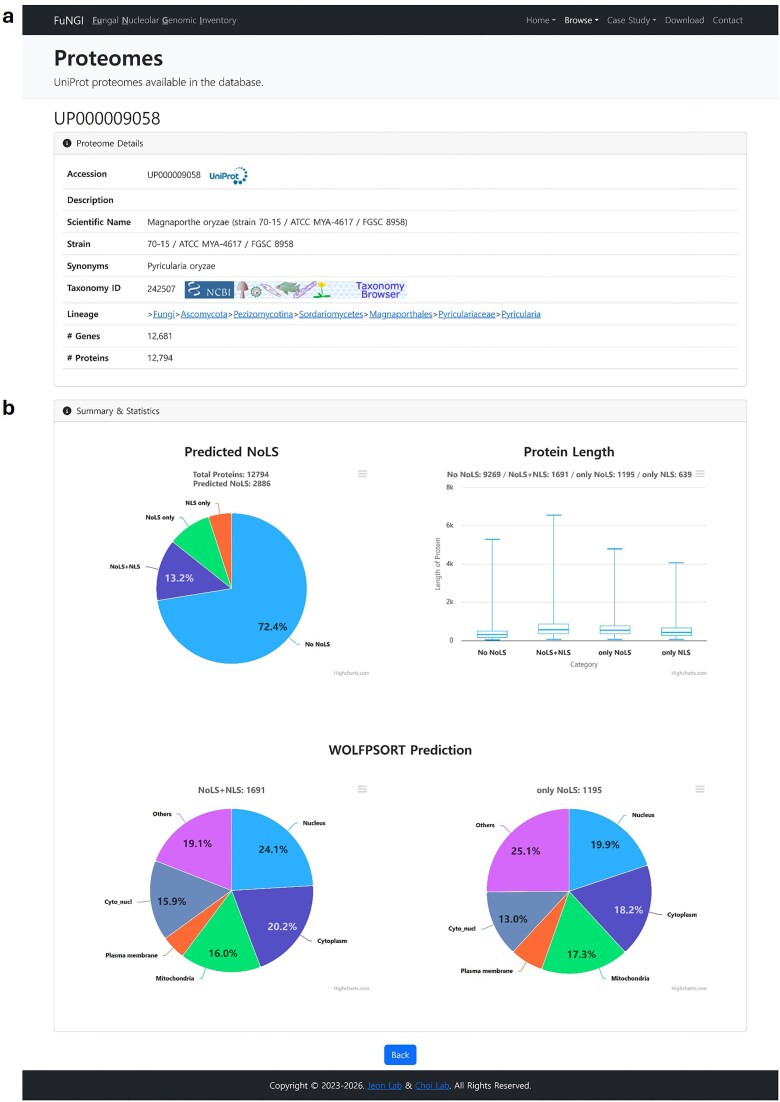
Snapshot of individual proteome information page in FuNGI. (a) Proteome Details: metadata for the selected fungal species, including UniProt accession, taxonomy, strain name, gene and protein counts, and hierarchical lineage. (b) Summary and Statistics: a pie chart showing the categorization of proteins by predicted nucleolar localization signals (NoLSs) and nuclear localization signals (NLSs); a boxplot showing the distribution of protein lengths across categories (No NoLS, NoLS + NLS, only NoLS, only NLS); and two pie charts showing subcellular localization predictions from WoLF PSORT for proteins containing NoLS + NLS and only NoLS.

Upon selecting a fungal species from either the Proteome browser or Taxonomy browser page, users are directed to a dedicated webpage containing detailed information about the selected proteome. This page is divided into two main sections: Proteome Details and Summary and Statistics (Fig. 4). The Proteome Details section (Fig. 4a) provides a comprehensive overview of the selected organism in a structured tabular format. Information includes the UniProt proteome ID (linked to the UniProt database), organism description, scientific name, strain, known synonyms, taxonomy ID (linked to the NCBI Taxonomy database), full taxonomy lineage, and the total number of genes and proteins associated with the species. The Summary and Statistics section (Fig. 4b) contains three interactive panels that highlight key characteristics of proteins predicted with nucleolar localization signals in the selected fungal species: (i) The Predicted NoLS panel: This panel presents a pie chart showing the distribution of proteins based on their predicted localization features. Categories include proteins with only NoLS, only NLS, both NoLS and NLS, and proteins lacking both NLS and NoLS. (ii) The Protein Length panel: This panel displays a boxplot illustrating the distribution of protein lengths across the same categories, proteins with only NoLS, only NLS, both NoLS and NLS, and neither. This allows users to compare the length profiles of proteins associated with NoLS versus those without NoLS. (iii) The WoLF PSORT Prediction panel: This panel contains two pie charts summarizing predicted subcellular localization. One chart represents proteins predicted to contain both NoLS and NLS, while the other represents proteins predicted to contain only NoLS. These visualizations provide insights into the dominant subcellular compartments associated with NoLS-positive proteins. Together, these interactive panels enable users to explore, compare, and interpret predicted nucleolar localization patterns within individual fungal proteomes, thereby supporting hypothesis generation and targeted analysis.

### Individual protein information

**Figure 5:**
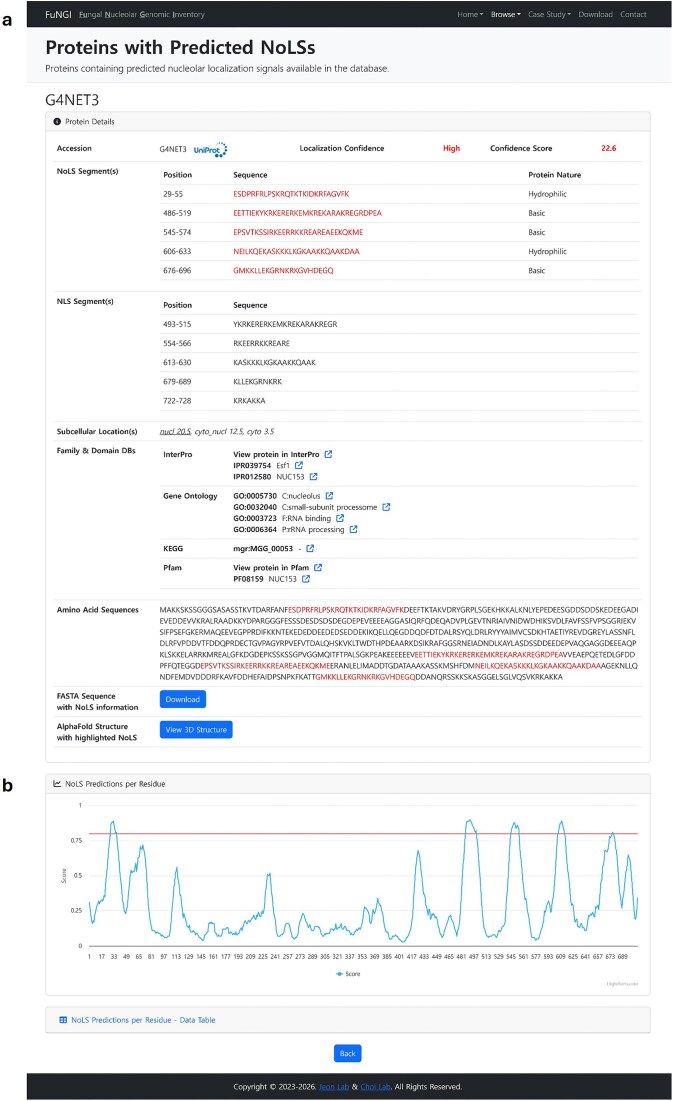
Snapshot of NoLS information for an individual protein of the selected organism in FuNGI. (a) Detailed information of the selected protein, including confidence rank, general information, and highlighted NoLS segments (in red). (b) NoD server prediction results displayed as a line graph, where the *x*-axis represents individual amino acid positions and the *y*-axis shows the predicted average score output of the artificial neural network (ANN) across eight successive windows. A NoLS segment is predicted when the average ANN score for eight successive windows is ≥0.8 (indicated by the red threshold line).

Upon clicking a UniProt accession number from the predicted NoLS-containing protein list of a selected fungal species, users are directed to a dedicated webpage that provides detailed information about the selected protein. This page is organized into two main sections: Protein Details and NoLS Predictions per Residue (Fig. 5). The Protein Details section presents comprehensive information about the selected protein (Fig. 5a). This includes the UniProt accession number, nucleolar localization confidence rank, computed confidence score, positions and sequences of predicted NoLS segments, along with their dominant amino acid composition; and positions and sequences of predicted NLS segments. Additionally, WoLF PSORT subcellular localization predictions are provided, along with protein family and domain annotations linked to external databases such as InterPro, Gene Ontology, KEGG, and Pfam. The full-length amino acid sequence is shown with predicted NoLS segments highlighted in red. Users can download the annotated sequence in FASTA format. An interactive 3D protein structure, predicted by AlphaFold, is also available through the integrated NGL Viewer, with NoLS regions visually highlighted for structural context, where users can also download the AlphaFold model in PDB format. The NoLS Predictions per Residue section (Fig. 5b) presents a graphical visualization of pre-computed nucleolar localization signal (NoLS) predictions at the residue level, generated using the NoD server. This interactive plot displays the NoD prediction scores across the full length of the protein sequence. Segments with scores exceeding the predefined threshold of 0.80 are identified as predicted NoLSs and are visually highlighted above a red reference line. This graphical output allows users to easily pinpoint regions within the protein that are likely to contribute to nucleolar localization. In addition to the graphical display, a collapsible data table lists the residue-level NoD prediction scores for in-depth analysis. Together, these sections provide a detailed and user-interactive view of individual proteins containing predicted NoLSs, supporting in-depth exploration of their sequence features, predicted localization behaviour, and structural context.

### Case study for *Magnaporthe oryzae* and validation of database entries

To evaluate the reliability of FuNGI predictions, we conducted a case study using *M. oryzae*, a filamentous fungus that causes rice blast disease and serves as a well-established model organism for plant-pathogen interaction studies. Its genetic tractability and extensive experimental background make it an ideal candidate for initial validation. Among the 12 794 protein-coding sequences in the *M. oryzae* genome, 2886 proteins (22.56%) were predicted to contain nucleolar localization signal (NoLS) segments. Of these NoLS-positive proteins, 1 195 (9.34%) lacked predicted nuclear localization signals (NLSs) (NoLS – NLS), while 1 691 (13.22%) were predicted to contain both NoLS and NLS segments (NoLS + NLS). Subcellular localization analysis using WoLF PSORT showed that 67.55% of the NoLS + NLS proteins were predicted to localize to the nucleus. Interestingly, 38.08% of the NoLS – NLS proteins were also predicted to localize to the nucleus, despite lacking canonical NLS motifs. These findings suggest that some NoLS-positive proteins may depend on interacting partners for nucleolar import or require specific cellular conditions for nucleolar targeting. The presence of a NoLS alone may not be sufficient for stable nucleolar localization, emphasizing the value of integrating multiple predictive features. To experimentally validate FuNGI predictions, five NoLS-positive proteins from *M. oryzae* were randomly selected; four ranked with high confidence and one with medium confidence (Table 1). To generate fluorescent fusion constructs, the coding sequences (excluding stop codons) of MoNOP58, MoRTT109, MoRRP8, MoNOLH1, and MoJMJD2T^0^ were amplified from *M. oryzae* KJ201 cDNA and fused in-frame to the N-terminus of green fluorescent protein (GFP) using the double-joint PCR strategy. Each GFP fusion construct was cloned into a T&A vector and introduced into *M. oryzae* via stable transformation, enabling expression of the fusion proteins in fungal hyphae. As a nucleolar reference marker, MoNOP1 was similarly fused to red fluorescent protein (RFP) using the same cloning approach. The *M. oryzae* strain KJ201 was co-transformed with the MoNOP1-RFP construct and each of the GFP-tagged candidate constructs using protoplast-mediated transformation. Transformants were cultured in liquid complete medium, and hyphae were harvested for microscopy. Hyphae were mounted in sterile water and imaged using a widefield fluorescence microscope (Leica model, equipped with a DFC7000 T camera) with sequential acquisition of GFP (excitation: 488 nm) and RFP (excitation: 561 nm) fluorescence channels. Differential interference contrast (DIC) images were also captured to visualize hyphal morphology.

**Table 1:** List of *Magnaporthe oryzae* proteins predicted with NoLS and NLS used for experimental validation.

					Predictions^a^				
Sl.	Locus	Identifier	Protein name	Length	A	B	C	Score	Confidence
1	MGG_00053	G4NET3	NUC153 domain-containing protein	728	5	5	nucl	22.6	High
2	MGG_05969	G4N4I8	Histone acetyltransferase	579	1	1	nucl	12.1	Medium
3	MGG_07008	G4MPA1	Nucleolar protein 58	599	3	2	cyto	16.6	High
4	MGG_07132	G4MT55	Ribosomal RNA-processing protein 8	507	3	6	cyto	18.2	High
5	MGG_09186	G4MPD2	[Histone H3]-trimethyl-L-lysine(9) demethylase	1529	2	3	cyto	22.4	High

a The number of NoLSs predicted by NoD (A), the number of NLSs predicted by NLStradamus (B), and the top subcellular localization from WoLF PSORT (C). WoLF PSORT predictions were interpreted as follows: ‘nucl’ for nuclear and ‘cyto’ for cytosol.

**Figure 6:**
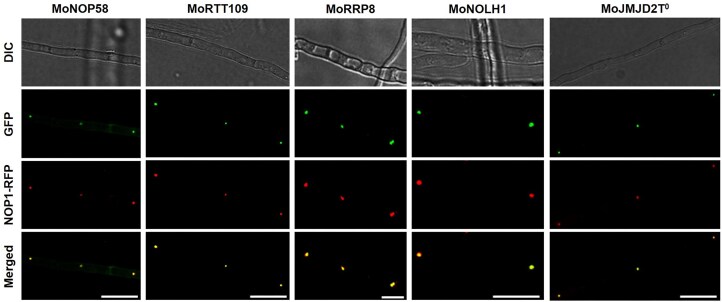
Co-localization of MoNOP1-RFP with candidate nucleolar proteins predicted by FuNGI in *Magnaporthe oryzae* hyphae. Fluorescence microscopy images of *M. oryzae* hyphae co-expressing the nucleolar marker MoNOP1-RFP and individual GFP-tagged candidate proteins predicted to localize to the nucleolus by FuNGI. From left to right: representative images of hyphae expressing MoNOP1-RFP with MoNOP58-GFP, MoRTT109-GFP, MoRRP8-GFP, MoNOLH1-GFP, and MoJMJD2T^0^-GFP. For each strain, differential interference contrast (DIC), green fluorescence (GFP), red fluorescence (MoNOP1-RFP), and merged images are shown. Yellow puncta in the merged panels indicate co-localization of candidate proteins with the nucleolar marker. Scale bars = 10 µm.

Representative images of hyphae co-expressing MoNOP1-RFP and each candidate protein-GFP fusion are shown in Fig. 6. In all five cases, GFP signals co-localized with MoNOP1-RFP within nucleolar regions, appearing as yellow spots in merged images. These findings provide experimental support for the predictive validity of FuNGI and demonstrate that its integrated scoring framework effectively prioritizes proteins with predicted nucleolar localization signals for functional characterization. FuNGI also identified multiple *M. oryzae* proteins associated with nucleolar ribosome biogenesis and rRNA modification complexes, illustrating its utility in capturing functionally relevant nucleolar candidates (Supplementary Table S4).

**Figure 7:**
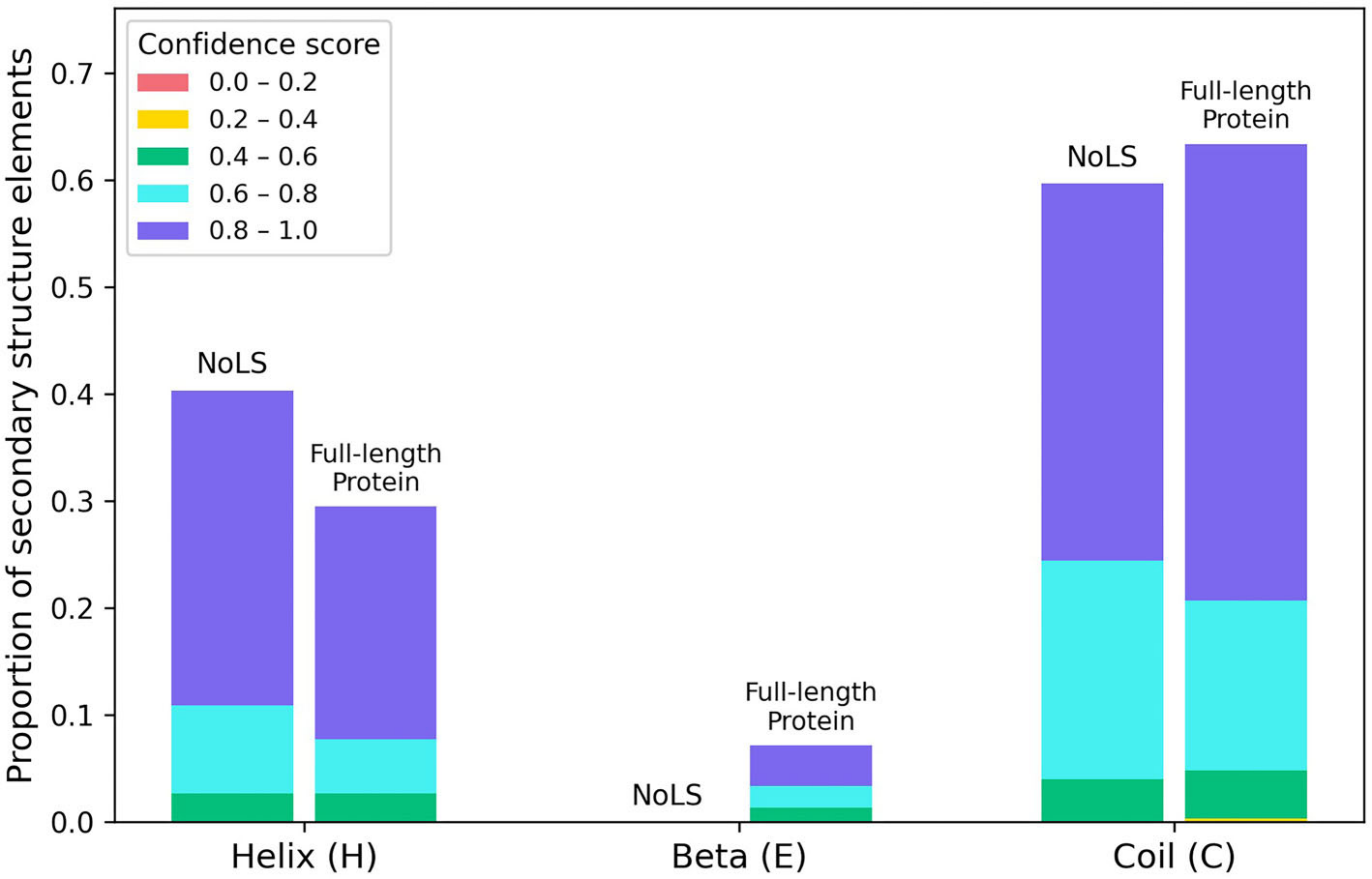
Proportion of secondary structure elements within NoLS regions and corresponding full-length sequences of five experimentally validated *M. oryzae* proteins. PSIPRED predictions were grouped by structural class (α-helix, β-strand, coil) and subdivided into confidence score intervals ([0.0–0.2] to [0.8–1.0]). NoLS regions occur predominantly within α-helices or coils, with β-strands not observed.

Additionally, the secondary structures of their NoLS regions were predicted using the PSIPRED (v4.01) [29]. The NoLS segments from the five validated *M. oryzae* proteins were predominantly located within α-helices or coils, whereas β-strand structures were not observed (Fig. 7).

### Ortholog-based analysis of conserved nucleolar proteins

To further illustrate the utility of FuNGI, we performed an ortholog-based analysis using two reference sets: 94 human nucleolar proteins curated from the Human Protein Atlas [30] and seven well-characterized *S. cerevisiae* nucleolar subunits representing the PeBoW complex (NOP7, ERB1, and YTM1) [31] and the box C/D small nucleolar ribonucleoprotein core complex (NOP1, NOP56, NOP58, and SNU13) [32]. Using a reciprocal best hit approach, orthologs were identified across 769 fungal proteomes by BLASTP (*E*-value of 1e-5), with identity and coverage thresholds of 25% and 40% for the human nucleolar proteins, and 45% and 70% for *S. cerevisiae* complex subunits (Supplementary Data Files S1–S4).

At the proteome level, substantial proportions of fungal orthologs derived from the 94 human nucleolar proteins contained predicted NoLSs and NLSs and were consistently assigned medium-to-high confidence scores in FuNGI (Supplementary Fig. S1). For example, *Schizosaccharomyces pombe* showed 16 orthologs containing predicted NoLSs among 20 detected orthologs (80.0%), with 14 also containing predicted NLSs and a mean confidence score of 14.995. Similarly, in *Aspergillus fumigatus*, 15 of 18 detected orthologs contained predicted NoLSs (83.3%), 13 also contained predicted NLSs, and the mean confidence score was 14.072.

Plant-associated pathogenic fungi also retained strong support: *Fusarium oxysporum* showed 15 orthologs containing predicted NoLSs among 19 detected orthologs (78.9%; mean confidence score = 14.432), *Ustilago maydis* contained 15 among 20 (75.0%; mean confidence score = 12.91), and *M. oryzae* contained 15 among 21 (71.4%; mean confidence score = 13.167). Together, these proteome-level patterns show that orthologs of human nucleolar proteins are broadly represented in FuNGI across diverse fungal proteomes, with strong support from predicted NoLS signals.

A more focused subunit-level analysis of the well-characterized yeast nucleolar complexes further showed that the confidence signals were heterogeneous across conserved nucleolar components rather than uniformly distributed. Several subunits, including NOP7, ERB1, NOP56, and NOP58, showed medium-to-high confidence scores across many fungal proteomes (Supplementary Fig. S2), whereas others, such as YTM1, NOP1, and SNU13, showed relatively low confidence patterns. These differences may reflect a combination of factors, including limitations of motif-based prediction, divergence in sequence features, and the possibility that some subunits depend more strongly on protein–protein interactions, complex assembly, post-translational modifications, or passive nuclear entry mechanism of small proteins.

Based on this analysis, we also implemented ortholog-based tutorial pages in FuNGI. These pages provide guided examples showing how conserved nucleolar proteins can be traced from model organisms to fungal orthologs and then directly connected to NoLS, NLS, and subcellular localization predictions in FuNGI. Therefore, these results indicate that FuNGI captures a substantial fraction of conserved nucleolar protein orthologs across fungal proteomes, highlighting its utility as a prioritization framework for identifying strongly supported nucleolar localization signals.

### Cross-species validation of NoD predictions using structural embeddings

To assess whether the NoD server trained on human proteins could be applied to fungal proteins, a structural similarity analysis was conducted using ESM Cambrian (ESM-C) embeddings [33]. Experimentally validated human NoLSs (HumanNoLS+; *n* = 46) and *M. oryzae* NoLSs (MoNoLS+; *n* = 14), derived from five validated proteins, were defined as the positive dataset. The negative dataset (MoNoLS−; *n* = 3 245) was constructed from *M. oryzae* proteins predicted to lack NoLS, NLS, and WoLF PSORT nuclear localization, by randomly generating windows that matched the length and positional distribution of MoNoLS+.

The high-dimensional embeddings of these sequences were projected into two dimensions using *t*-distributed stochastic neighbour embedding (*t*-SNE) [34], with a perplexity of 20. To statistically assess proximity, Euclidean mean distances between Human NoLS+ (*n* = 46) and MoNoLS+ (*n* = 14) were then calculated, compared with randomly sampled MoNoLS− (*n* = 14; matched to the number of MoNoLS+). A random sampling test (1000 iterations) showed that the average distance for MoNoLS+ was significantly lower than expected by chance (*P* = 0.013; Supplementary Fig. S3).

Since distances in the reduced space may not faithfully reflect the true structural similarity in high-dimensional space, machine learning models were further trained directly on the ESM-C embeddings. A dataset comprising 46 HumanNoLS+ and 14 MoNoLS+ as positives, together with 60 randomly sampled MoNoLS− (from 3 245 sequences) as negatives, was used for modelling. Stratified five-fold cross-validation was repeated 1000 times, generating independent assessments of model performance. Random Forest, Gradient Boosting (XGBoost), Support Vector Machines, and Logistic Regression all achieved high classification performance (Supplementary Table S5), reliably distinguishing HumanNoLS+ and MoNoLS+ from negatives.

These results indicate that the observed proximity is not an artefact of dimensionality reduction but rather reflects genuine features of the structural embeddings. At the motif level, *M. oryzae* NoLSs share conserved structural signals with human NoLSs, supporting the applicability of the human-trained NoD server to fungal proteins.

### Limitations of FuNGI

Although the presence of a predicted nucleolar localization signal (NoLS) provides useful evidence for potential nucleolar targeting, it does not guarantee nucleolar localization *in vivo*. Protein localization is influenced by multiple factors, including protein–protein interactions, post-translational modifications, cellular context, and dynamic nucleocytoplasmic trafficking.

In addition, the current framework does not distinguish localization within nucleolar subcompartments such as the FC, DFC, and GC, since such partitioning is more likely determined by functional context and molecular interactions than by sequence-based signal prediction alone. FuNGI should therefore be interpreted as a resource for prioritizing candidate fungal proteins with predicted nucleolar localization signals and for supporting comparative analyses and future experimental validation of nucleolar biology across fungi.

Furthermore, small proteins (generally smaller than ∼40 kDa) may enter the nucleus through passive diffusion without requiring a classical nuclear localization signal (NLS) [35]. This suggests that some nucleolar proteins may exhibit biologically relevant nuclear or nucleolar localization despite lacking identifiable targeting signals, and this possibility should be considered when interpreting prediction-based results.

## Conclusion

We present FuNGI, a dedicated knowledge base of fungal proteins containing predicted nucleolar localization signals. The database is built on a user-friendly web platform that supports browsing, searching, visualization, and data download. The current version includes annotated proteins containing predicted nucleolar localization signals from 769 fungal species, integrating NoLS and NLS predictions, subcellular localization data, and structural visualization. The comprehensive dataset spans diverse fungal phyla, offering insights into nucleolar diversity and evolution. Future developments will focus on expanding the database with additional fungal genomes, refining the prediction algorithms, and introducing new features such as comparative analysis tools and user-submitted validation data. We anticipate that FuNGI will serve as a valuable resource for researchers exploring the roles of predicted proteins containing nucleolar localization signals in fungal biology, pathogenesis, and evolution.

## Supplementary Material

baag023_Supplemental_Files

## Data Availability

Users can use any feature available in the database without registration or log-in. All data are available and freely accessible at https://fungi.toolbox.or.kr/. The ortholog mapping datasets generated in this study are provided as Supplementary Material (Files S1–S4).

## References

[1] Dubois ML, Boisvert FM. The nucleolus: structure and function. In: Bazett-Jones D, Dellaire G (eds), The Functional Nucleus. Cham: Springer, 2016, 29–49. 10.1007/978-3-319-38882-3_2

[2] Liu Y, Li Y, Lu X. Regulators in the DNA damage response. Arch Biochem Biophys. 2016;594:18–25. 10.1016/j.abb.2016.02.018 26882840

[3] Tiku V, Antebi A. Nucleolar function in lifespan regulation. Trends Cell Biol. 2018;28:662–72. 10.1016/j.tcb.2018.03.007 29779866

[4] de Melo IS, Jimenez-Nuñez MD, Iglesias C et al. NOA36 protein contains a highly conserved nucleolar localization signal capable of directing functional proteins to the nucleolus in mammalian cells. PLoS One. 2013;8:e59065. 10.1371/journal.pone.0059065 23516598 PMC3596294

[5] Roche B, Arcangioli B, Martienssen R. New roles for Dicer in the nucleolus and its relevance to cancer. Cell Cycle. 2017;16:1643–53. 10.1080/15384101.2017.1361568 28846478 PMC5616133

[6] Lindström MS, Jurada D, Bursac S et al. Nucleolus as an emerging hub in maintenance of genome stability and cancer pathogenesis. Oncogene. 2018;37:2351–66. 10.1038/s41388-017-0121-z 29429989 PMC5931986

[7] Leung AK, Lamond AI. The dynamics of the nucleolus. Crit Rev Eukaryot Gene Expr. 2003;13:39–54. 10.1615/CritRevEukaryotGeneExpr.v13.i1.40 12839096

[8] Muñoz-Díaz E, Sáez-Vásquez J. Nuclear dynamics: formation of bodies and trafficking in plant nuclei. Front Plant Sci. 2022;13:984163. 10.3389/fpls.2022.984163 36082296 PMC9445803

[9] Lu J, Wu T, Zhang B et al. Types of nuclear localization signals and mechanisms of protein import into the nucleus. Cell Commun Signal. 2021;19:60. 10.1186/s12964-021-00741-y 34022911 PMC8140498

[10] Martin RM, Ter-Avetisyan G, Herce HD et al. Principles of protein targeting to the nucleolus. Nucleus. 2015;6:314–25. 10.1080/19491034.2015.1079680 26280391 PMC4615656

[11] Carmo-Fonseca M, Mendes-Soares L, Campos I. To be or not to be in the nucleolus. Nat Cell Biol. 2000;2:E107–12. 10.1038/35014078 10854340

[12] Scott MS, Boisvert FM, McDowall MD et al. Characterization and prediction of protein nucleolar localization sequences. Nucleic Acids Res. 2010;38:7388–99. 10.1093/nar/gkq653 20663773 PMC2995072

[13] Scott MS, Troshin PV, Barton GJ. NoD: a nucleolar localization sequence detector for eukaryotic and viral proteins. BMC Bioinf. 2011;12:317. 10.1186/1471-2105-12-317 PMC3166288 21812952

[14] Bauer NC, Doetsch PW, Corbett AH. Mechanisms regulating protein localization. Traffic. 2015;16:1039–61. 10.1111/tra.12310 26172624

[15] Shrikondawar AN, Chennoju K, Ghosh DK et al. Identification and functional characterization of the nuclear and nucleolar localization signals in the intrinsically disordered region of nucleomethylin. J Cell Physiol. 2024;239:e31433. 10.1002/jcp.31433 39245872

[16] Yang CP, Chiang CW, Chen CH et al. Identification and characterization of nuclear and nucleolar localization signals in 58-kDa microspherule protein (MSP58). J Biomed Sci. 2015;22:33. 10.1186/s12929-015-0136-0 25981436 PMC4434885

[17] Leung AK, Trinkle-Mulcahy L, Lam YW et al. NOPdb: nucleolar proteome database. Nucleic Acids Res. 2006;34:D218–20. 10.1093/nar/gkj004 16381850 PMC1347367

[18] Lee SH, Dubey N, Jeon J. The unknown within the known: nucleolus, understudied compartment in the filamentous fungi. Mycobiology. 2024;52:214–21. 10.1080/12298093.2024.2379623 39445133 PMC11494718

[19] Bhabhra R, Zhao W, Rhodes JC et al. Nucleolar localization of *Aspergillus fumigatus* CgrA is temperature-dependent. Fung Genet Biol. 2006;43:1–7. 10.1016/j.fgb.2005.07.005 16314125

[20] UniProt Consortium . UniProt: the universal protein knowledgebase in 2021. Nucleic Acids Res. 2021;49:D480–89. 10.1093/nar/gkaa1100 33237286 PMC7778908

[21] Ba ANN, Pogoutse A, Provart N et al. NLStradamus: a simple Hidden Markov Model for nuclear localization signal prediction. BMC Bioinf. 2009;10:202. 10.1186/1471-2105-10-202 PMC2711084 19563654

[22] Horton P, Park KJ, Obayashi T et al. WoLF PSORT: protein localization predictor. Nucleic Acids Res. 2007;35:W585–87. 10.1093/nar/gkm259 17517783 PMC1933216

[23] Kosugi S, Hasebe M, Tomita M et al. Systematic identification of cell cycle-dependent yeast nucleocytoplasmic shuttling proteins by prediction of composite motifs. Proc Natl Acad Sci. 2009;106:10171–76. 10.1073/pnas.0900604106 19520826 PMC2695404

[24] Nair R, Carter P, Rost B. NLSdb: database of nuclear localization signals. Nucleic Acids Res. 2003;31:397–99. 10.1093/nar/gkg001 12520032 PMC165448

[25] Lin JR, Hu J. SeqNLS: nuclear localization signal prediction based on frequent pattern mining and linear motif scoring. PLoS One. 2013;8:e76864. 10.1371/journal.pone.0076864 24204689 PMC3812174

[26] Staub E, Mackowiak S, Vingron M. An inventory of yeast proteins associated with nucleolar and ribosomal components. Genome Biol. 2006;7:R98. 10.1186/gb-2006-7-10-r98 17067374 PMC1794573

[27] Rose AS, Hildebrand PW. NGL Viewer: a web application for molecular visualization. Nucleic Acids Res. 2015;43:W576–79. 10.1093/nar/gkv402 25925569 PMC4489237

[28] Varadi M, Anyango S, Deshpande M et al. AlphaFold Protein Structure Database: massively expanding the structural coverage of protein-sequence space with high-accuracy models. Nucleic Acids Res. 2022;50:D439–44. 10.1093/nar/gkab1061 34791371 PMC8728224

[29] Buchan DW, Jones DT. The PSIPRED protein analysis workbench: 20 years on. Nucleic Acids Res. 2019;47:W402–W407. 10.1093/nar/gkz297 31251384 PMC6602445

[30] Stenström L, Mahdessian D, Gnann C et al. Mapping the nucleolar proteome reveals a spatiotemporal organization related to intrinsic protein disorder. Mol Syst Biol. 2020;16:e9469. 10.15252/msb.20209469 32744794 PMC7397901

[31] Hölzel M, Rohrmoser M, Schlee M et al. Mammalian WDR12 is a novel member of the Pes1-Bop1 complex and is required for ribosome biogenesis and cell proliferation. J Cell Biol. 2005;170:367–78. 10.1083/jcb.200501141 16043514 PMC2171466

[32] Lafontaine DL, Tollervey D. Synthesis and assembly of the box C+D small nucleolar RNPs. Mol Cell Biol. 2000;20:2650–59. 10.1128/MCB.20.8.2650-2659.2000 10733567 PMC85480

[33] ESM Team . ESM Cambrian: revealing the mysteries of proteins with unsupervised learning. Evolutionary Scale. 2024. https://evolutionaryscale.ai/blog/esm-cambrian (9 August 2025, date last accessed).

[34] Maaten LV, Hinton G. Visualizing data using t-SNE. J Mach Learn Res. 2008;9:2579–605.

[35] Makio T, Wozniak RW. Passive diffusion through nuclear pore complexes regulates levels of the yeast SAGA and SLIK coactivator complexes. J Cell Sci. 2020;133:jcs237156. 10.1242/jcs.237156 32051285

