# Supplementary material for: FuNGI: Fungal Nucleolar Genomic Inventory—a comprehensive database of fungal proteins with predicted nucleolar localization signals
Source: Database (Oxford). 2026 May 19;2026:baag023. doi: 10.1093/database/baag023 (PMC13184608; doi:10.1093/database/baag023)
Supplement: baag023_Supplemental_Files [file baag023_supplemental_files.zip › Supplementary_Information_clean.docx]

**Supplementary Information**

**FuNGI: Fungal Nucleolar Genomic Inventory –A comprehensive database of fungal proteins with predicted nucleolar localization signals**

Gnanendra Shanmugam^1†^, Chaewon Kim^2†^, Surajit De Mandal^1^, Kim Minji^1^_,_ Song Hee Lee^1^, Jaeyoung Choi^2,3*^, and Junhyun Jeon^1,4*^

^1^Department of Biotechnology, College of Life and Applied Sciences, Yeungnam University, Gyeongsan, Gyeongbuk, 38541, Republic of Korea

^2^Department of Convergent Biotechnology and Advanced Materials Science, College of Life Sciences, Kyung Hee University, Yongin, 17104, Republic of Korea

^3^BK21 Interdisciplinary Program in IT-Bio Convergence System, Kyung Hee University, Yongin 17104, Republic of Korea

^4^Phytobiome Engineering Research Laboratory, Yeungnam University, Gyeongsan, Gyeongbuk, 38541, Republic of Korea

^†^These authors contributed equally to the work.

*Correspondence to: Jaeyoung Choi & Junhyun Jeon

Phone: +82-31-201-2695 (JC) & +82-2-810-3030 (JJ)

Fax: +82-53-810-4769 (JJ)

E-mail: [jaeyoung.choi@khu.ac.kr](mailto:jaeyoung.choi@khu.ac.kr) (JC) & [jjeon@yu.ac.kr](mailto:jjeon@ynu.ac.kr) (JJ)

**List of Supplementary Figures**

**Supplementary Figure S1.** Proteome-level overview of fungal orthologs of 94 human nucleolar proteins in FuNGI. Scatter plot showing the proportion of detected orthologs with predicted NLSs (x-axis) and predicted NoLSs (y-axis) in each fungal proteome. Each dot represents one proteome, and the color scale indicates the mean FuNGI confidence score of the corresponding orthologs.

**Supplementary Figure S2.** Heatmap showing FuNGI confidence scores for detected orthologs of representative *Saccharomyces cerevisiae* nucleolar complex subunits across 769 proteomes. Columns correspond to the subunits from the PeBoW complex (NOP7, ERB1, YTM1) and the box C/D small nucleolar ribonucleoprotein (snoRNP) core complex (NOP1, NOP56, NOP58, SNU13). Rows represent fungal proteomes. Cell colors indicate FuNGI confidence scores, whereas gray cells indicate that no ortholog was detected for the corresponding subunit in that proteome.

**Supplementary Figure S3.** Histogram of Euclidean mean distances between experimentally validated nucleolar sequences from human (HumanNoLS+; n=46) and *Magnaporthe oryzae* (MoNoLS+; n=14), compared with randomly sampled MoNoLS– (n=14; matched to the number of MoNoLS+) in *t*-SNE reduced embedding space. The random sampling was repeated 1,000 times to generate a null distribution (blue bars). The red dashed line indicates the average distance for MoNoLS+, which was significantly lower than expected by chance (p = 0.013), supporting their proximity to human nucleolar proteins in embedding space.

**List of Supplementary Tables**

**Supplementary Table S1.** Comparison of NLS prediction efficiency of different algorithms on the experimentally validated nucleolar proteins from human that have been used to develop the NoD server.

**Supplementary Table S2.** Comparison of predicted NoLS, NLS and subcellular location results of experimentally validated nucleolar protein sequence from human and yeast for the implementation of confidence ranking. The percentage of proteins predicted in each class is given in parenthesis.

**Supplementary Table S3**. Confidence scores and categories for the 43 human and 140 yeast validated protein sequences.

**Supplementary Table S4**. Examples of proteins associated with nucleolar ribosome biogenesis and rRNA modification complexes identified in *Magnaporthe oryzae* using FuNGI.

**Supplementary Table S5.** Classification performance of four machine learning algorithms (Random Forest, Gradient Boosting (XGBoost), Support Vector Machines (SVM), and Logistic Regression (LR)) trained on ESM C embeddings to distinguish positives (experimentally validated human and *Magnaporthe oryzae* NoLSs) from *M. oryzae* negative datasets. AUC, precision, recall, F1-score and accuracy are represented as mean ± standard deviation over 1,000 iterations of stratified 5-fold cross-validation.

**
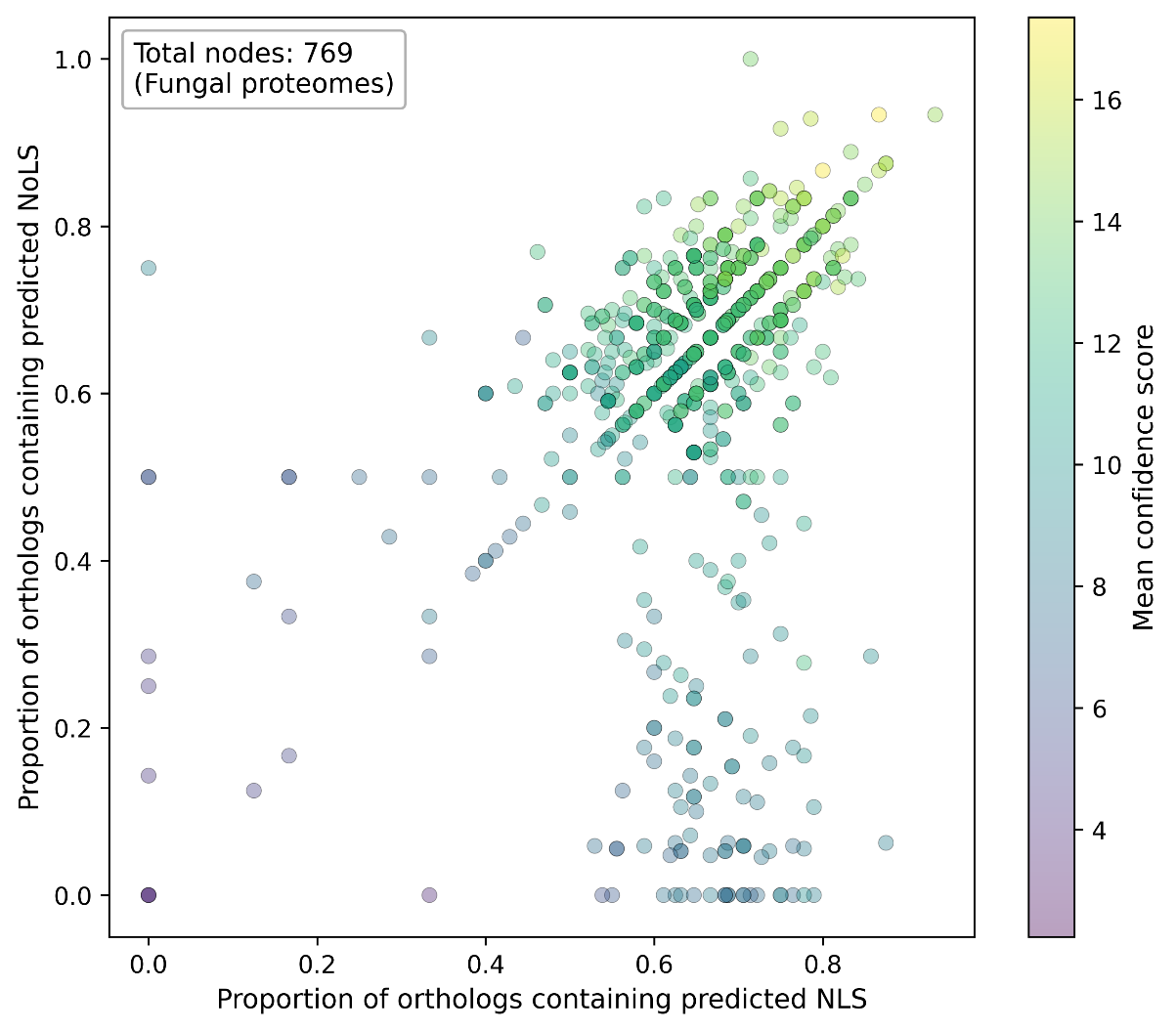
**

**Supplementary Figure S1.** Proteome-level overview of fungal orthologs of 94 human nucleolar proteins in FuNGI. Scatter plot showing the proportion of detected orthologs with predicted NLSs (x-axis) and predicted NoLSs (y-axis) in each fungal proteome. Each dot represents one proteome, and the color scale indicates the mean FuNGI confidence score of the corresponding orthologs.

**
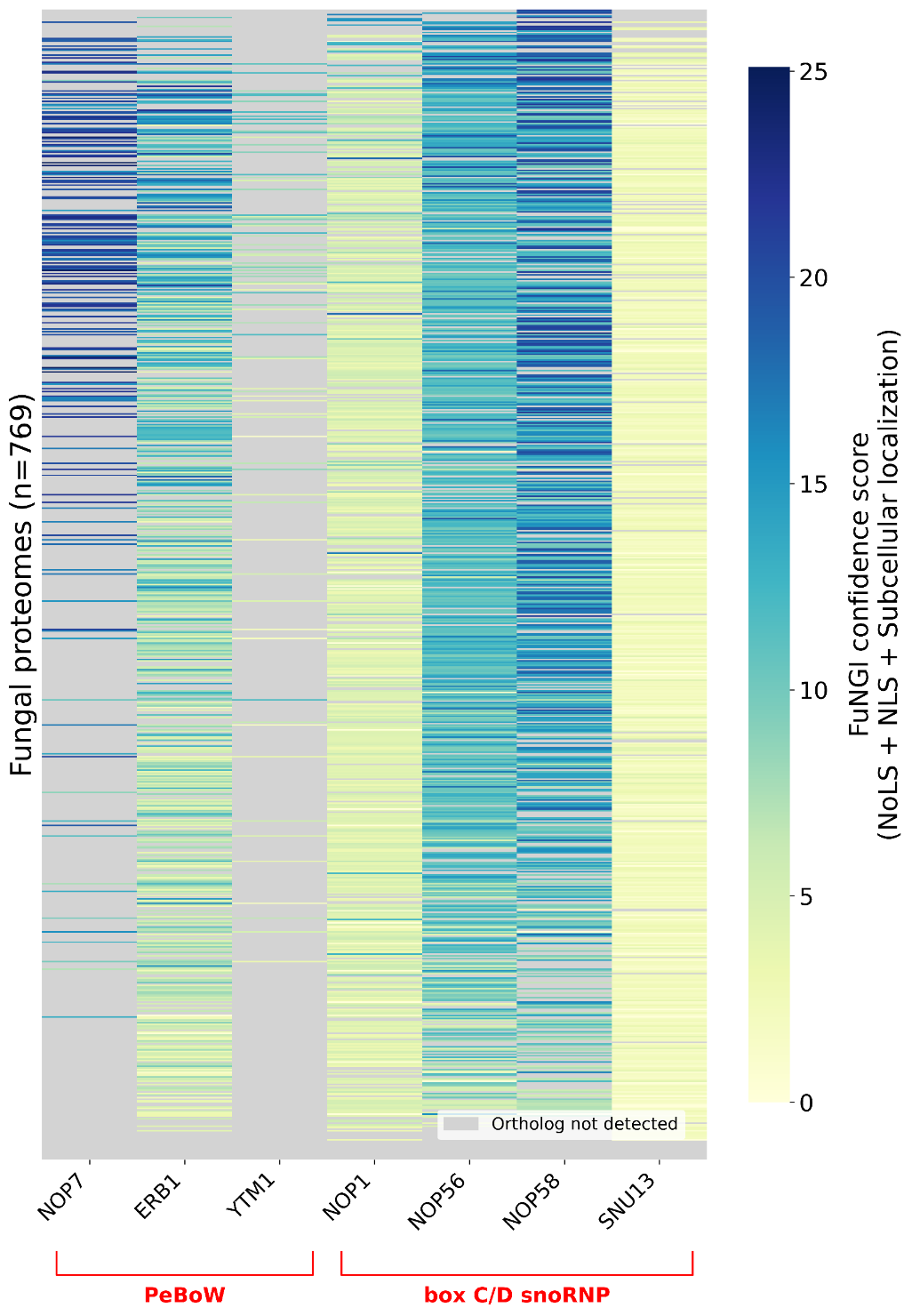
**

**Supplementary Figure S2.** Heatmap showing FuNGI confidence scores for detected orthologs of representative *Saccharomyces cerevisiae* nucleolar complex subunits across 769 proteomes. Columns correspond to the subunits from the PeBoW complex (NOP7, ERB1, YTM1) and the box C/D small nucleolar ribonucleoprotein (snoRNP) core complex (NOP1, NOP56, NOP58, SNU13). Rows represent fungal proteomes. Cell colors indicate FuNGI confidence scores, whereas gray cells indicate that no ortholog was detected for the corresponding subunit in that proteome.

**
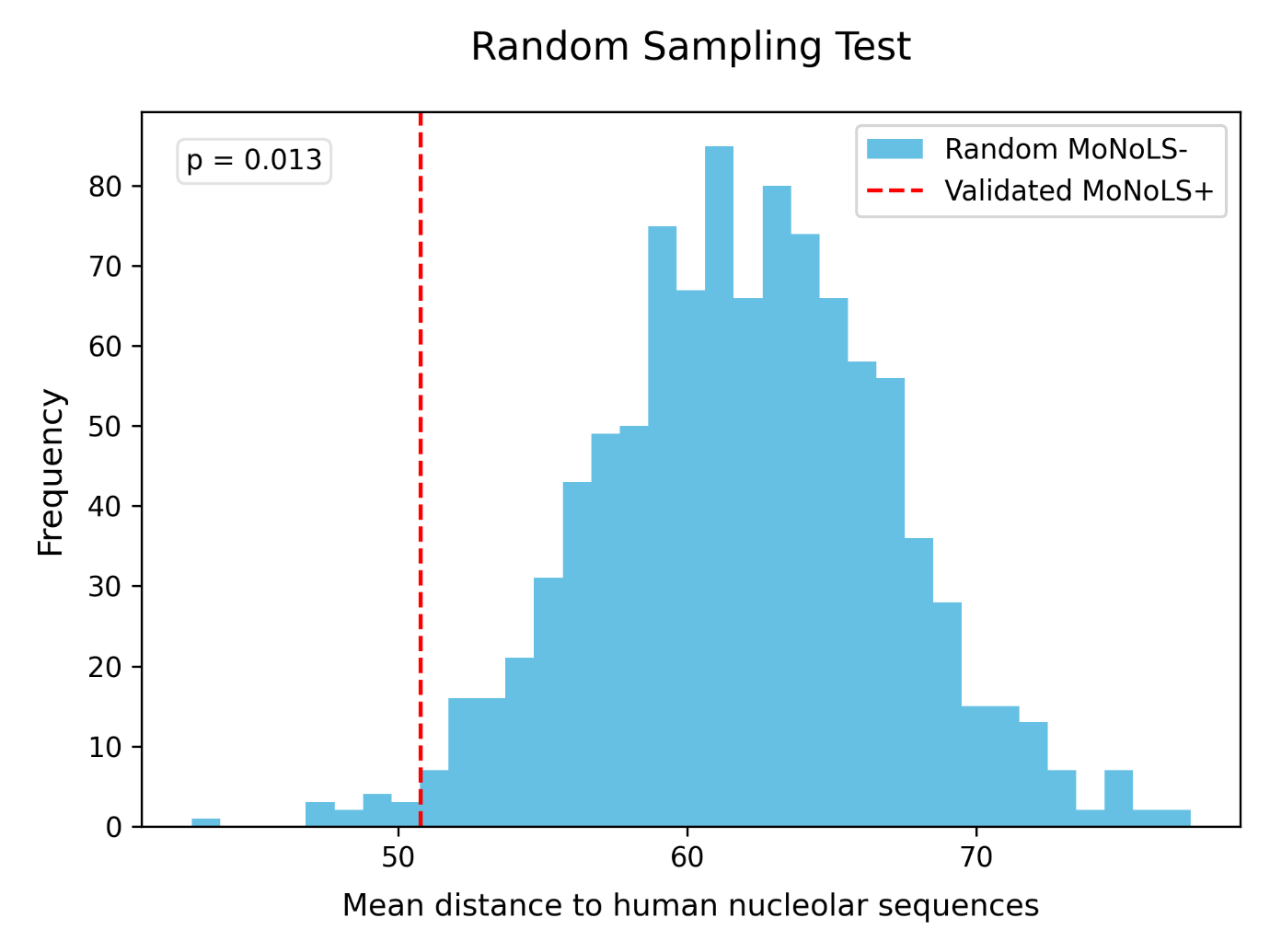
**

**Supplementary Figure S3.** Histogram of Euclidean mean distances between experimentally validated nucleolar sequences from human (HumanNoLS+; n=46) and *Magnaporthe oryzae* (MoNoLS+; n=14), compared with randomly sampled MoNoLS– (n=14; matched to the number of MoNoLS+) in *t*-SNE reduced embedding space. The random sampling was repeated 1,000 times to generate a null distribution (blue bars). The red dashed line indicates the average distance for MoNoLS+, which was significantly lower than expected by chance (p = 0.013), supporting their proximity to human nucleolar proteins in embedding space.

**Supplementary Table S1.** Comparison of NLS prediction efficiency of different algorithms on the experimentally validated nucleolar proteins from human that have been used to develop the NoD server.

| **Sl.** | **Accession** | **Symbol** | **cNLS_Mapper** | **NLStrdamus** | **NLSdb** | **seqNLS** |
| --- | --- | --- | --- | --- | --- | --- |
| 1 | NP_001012270 | BIRC5 | Yes | Yes | Yes | No |
| 2 | NP_006161 | NOP2 | Yes | Yes | Yes | No |
| 3 | NP_005336 | HSPA1A | No | Yes | Yes | No |
| 4 | NP_937862 | ING1b | Yes | Yes | Yes | No |
| 5 | NP_005238 | FGF3 | No | Yes | Yes | No |
| 6 | NP_006618 | POP4 | No | Yes | Yes | No |
| 7 | NP_945316 | PTHLH | Yes | Yes | No | No |
| 8 | NP_003778 | NOL4 | Yes | No | Yes | No |
| 9 | NP_001002 | RPS7 | No | Yes | No | No |
| 10 | NP_001034800 | DEDD | Yes | Yes | Yes | No |
| 11 | NP_001091059 | RPP38 | Yes | Yes | No | No |
| 12 | NP_478102 | CDKN2A | No | Yes | No | No |
| 13 | NP_003133 | SSB | Yes | Yes | No | No |
| 14 | NP_005560 | LIMK2 | No | Yes | No | No |
| 15 | NP_001997 | FGF2 | No | Yes | No | No |
| 16 | NP_477352 | PI4KA | Yes | No | No | No |
| 17 | NP_002383 | MDM2 | Yes | No | Yes | No |
| 18 | NP_003945 | MAP3K14 | Yes | Yes | No | No |
| 19 | NP_078908 | SAP30L | Yes | No | Yes | No |
| 20 | NP_951038 | MDFIC | No | Yes | No | No |
| 21 | NP_848927 | MTDH | Yes | Yes | Yes | No |
| 22 | NP_078805 | CDC73 | Yes | No | Yes | No |
| 23 | NP_078905 | MLF1IP | Yes | Yes | Yes | No |
| 24 | NP_060239 | G2E3 | No | No | No | No |
| 25 | NP_077289 | NOL12 | Yes | Yes | Yes | No |
| 26 | NP_039252 | NRG1 | No | Yes | No | No |
| 27 | NP_055318 | UTP20 | Yes | Yes | Yes | No |
| 28 | NP_849193 | STT3B | Yes | Yes | No | Yes |
| 29 | NP_068810 | RELA | No | No | No | No |
| 30 | NP_112578 | INO80B | Yes | Yes | No | No |
| 31 | AAB60345 | L1 ORF2 | Yes | Yes | No | No |
| 32 | AAH01024 | GNL3 | Yes | Yes | Yes | No |
| 33 | NP_002511 | NPM1 | Yes | No | Yes | Yes |
| 34 | NP_937983 | TERT | Yes | Yes | Yes | No |
| 35 | NP_003277 | TOP1 | Yes | Yes | Yes | No |
| 36 | NP_796375 | MIDN | No | Yes | Yes | No |
| 37 | NP_004851 | FXR2 | No | Yes | Yes | No |
| 38 | NP_000347 | TCOF1 | Yes | Yes | Yes | No |
| 39 | NP_004695 | RRP9 | Yes | Yes | Yes | No |
| 40 | NP_150241 | PML | Yes | No | Yes | No |
| 41 | NP_061940 | GNL3L | Yes | Yes | No | No |
| 42 | NP_004251 | RECQL4 | No | Yes | No | No |
| 43 | NP_068778 | PPP1R11 | Yes | Yes | No | No |

**Supplementary Table S2.** Comparison of predicted NoLS, NLS and subcellular location results of experimentally validated nucleolar protein sequence from human and yeast for the implementation of confidence ranking. The percentage of proteins predicted in each class is given in parenthesis.

| **Predictions^*^** | | | **Human** | **Yeast** | **Confidence**  **Rank** |
| --- | --- | --- | --- | --- | --- |
| **A** | **B** | **C** |  |  |  |
| Y | Y | nucl | 21 (48.83%) | 74 (52.85%) | High |
| Y | N | nucl | 5 (11.62%) | 7 (5.00 %) |  |
| Y | Y | cyto | 2 (4.65%) | 6 (4.28%) | Medium |
| Y | N | cyto | 0 | 2 (1.42%) |  |
| Y | Y | others | 8 (18.60%) | 7 (5.00%) | Low |
| Y | N | others | 0 | 0 (0.71%) |  |
| N | Y | nucl | 2 (4.65%) | 11 (7.85%) | - |
| N | Y | cyto | 0 | 1 (0.71%) |  |
| N | Y | others | 2 (4.65%) | 1 (0.71%) |  |
| N | N | nucl | 3 (6.97%) | 20 (14.58%) |  |
| N | N | cyto | 0 | 9 (6.42%) |  |
| N | N | others | 0 | 2 (1.42%) |  |
| **The Number of Protein Sequences** | | | **43** | **140** |  |

^*^NoD (A), NLStradamus (B), and WoLF PSORT (C). Predictions from NoD and NLStradamus were marked “Y” for positive and “N” for negative. WoLF PSORT predictions were interpreted as follows: “nucl” for nuclear, “cyto” for cytosol, and “others” for subcellular locations other than nucleus and cytoplasm.

**Supplementary Table S3**. Confidence scores and categories for the 43 human and 140 yeast validated protein sequences.

| **Species** | **Accession** | **Predictions^*^** | | | **Confidence Score** | **Confidence Rank** |
| --- | --- | --- | --- | --- | --- | --- |
|  |  | **A** | **B** | **C** |  |  |
| Human | NP_001012270.1 | Y | Y | others | 17 | High |
| Human | NP_006161.2 | Y | Y | nucl | 39 | High |
| Human | NP_005336.3 | Y | Y | cyto | 22 | High |
| Human | NP_937862.1 | Y | Y | nucl | 36 | High |
| Human | NP_005238.1 | Y | Y | nucl | 19 | High |
| Human | NP_006618.1 | Y | Y | nucl | 32.5 | High |
| Human | NP_945316.1 | Y | Y | nucl | 21.333 | High |
| Human | NP_003778.2 | Y | N | nucl | 30.5 | High |
| Human | NP_001002.1 | Y | Y | cyto | 21.833 | High |
| Human | NP_001034800.1 | N | Y | nucl | 21.5 | High |
| Human | NP_001091059.1 | Y | Y | nucl | 25.5 | High |
| Human | NP_478102.2 | Y | Y | other | 8 | Low |
| Human | NP_003133.1 | Y | Y | nucl | 23.5 | High |
| Human | NP_005560.1 | Y | Y | nucl | 26 | High |
| Human | NP_001997.5 | Y | Y | nucl | 20.5 | High |
| Human | NP_477352.3 | N | N | nucl | 8 | Low |
| Human | NP_002383.2 | Y | N | nucl | 23 | High |
| Human | NP_003945.2 | Y | Y | nucl | 28.5 | High |
| Human | NP_078908.1 | Y | N | nucl | 25 | High |
| Human | NP_951038.1 | Y | Y | others | 14 | Medium |
| Human | NP_848927.2 | Y | Y | nucl | 28.5 | High |
| Human | NP_078805.3 | N | N | nucl | 13.5 | Medium |
| Human | NP_078905.2 | Y | Y | nucl | 37 | High |
| Human | NP_060239.2 | Y | N | nucl | 24 | High |
| Human | NP_077289.1 | Y | Y | nucl | 38.5 | High |
| Human | NP_039252.2 | Y | Y | nucl | 22.5 | High |
| Human | NP_055318.2 | Y | Y | nucl | 33.5 | High |
| Human | NP_849193.1 | Y | Y | others | 8 | Low |
| Human | NP_068810.3 | N | N | nucl | 18.5 | High |
| Human | NP_112578.2 | Y | Y | others | 21.5 | High |
| Human | AAB60345.1 | Y | Y | others | 19 | High |
| Human | AAH01024.1 | Y | Y | nucl | 35 | High |
| Human | NP_002511.1 | N | Y | nucl | 20 | High |
| Human | NP_937983.2 | N | Y | others | 3 | Low |
| Human | NP_003277.1 | Y | Y | nucl | 40 | High |
| Human | NP_796375.3 | Y | Y | nucl | 32 | High |
| Human | NP_004851.2 | Y | Y | nucl | 39.5 | High |
| Human | NP_000347.2 | Y | Y | others | 28.666 | High |
| Human | NP_004695.1 | N | Y | others | 9 | Low |
| Human | NP_150241.2 | Y | N | nucl | 28.5 | High |
| Human | NP_061940.1 | Y | Y | nucl | 32.5 | High |
| Human | NP_004251.4 | Y | Y | others | 17 | High |
| Human | NP_068778.1 | Y | Y | nucl | 30.5 | High |
| Yeast | P07271 | Y | Y | others | 28 | High |
| Yeast | P06700 | N | Y | nucl | 22 | High |
| Yeast | P25443 | Y | Y | cyto | 17 | High |
| Yeast | P45818 | Y | Y | nucl | 29.5 | High |
| Yeast | P25808 | Y | Y | nucl | 31.5 | High |
| Yeast | P40693 | Y | Y | nucl | 30 | High |
| Yeast | P48561 | Y | Y | nucl | 38 | High |
| Yeast | P32892 | Y | Y | nucl | 42.5 | High |
| Yeast | P0CX37 | Y | Y | cyto | 25.5 | High |
| Yeast | O13516 | N | N | nucl | 16 | High |
| Yeast | P26786 | N | Y | cyto | 16.833 | High |
| Yeast | Q01080 | Y | N | nucl | 19 | High |
| Yeast | Q12149 | Y | Y | nucl | 34 | High |
| Yeast | P06367 | Y | N | cyto | 12.5 | Medium |
| Yeast | P20448 | Y | Y | nucl | 38.5 | High |
| Yeast | Q04660 | Y | Y | nucl | 31 | High |
| Yeast | P33322 | Y | Y | nucl | 29.5 | High |
| Yeast | Q12389 | Y | Y | nucl | 42 | High |
| Yeast | P25586 | Y | Y | nucl | 32 | High |
| Yeast | Q12176 | Y | Y | nucl | 40.5 | High |
| Yeast | Q12136 | Y | Y | nucl | 35 | High |
| Yeast | Q12052 | Y | Y | others | 23.833 | High |
| Yeast | Q05024 | Y | Y | nucl | 32.5 | High |
| Yeast | Q12220 | Y | Y | nucl | 19 | High |
| Yeast | P20447 | Y | Y | nucl | 32 | High |
| Yeast | Q05498 | Y | Y | nucl | 29.5 | High |
| Yeast | Q12035 | Y | Y | nucl | 33.5 | High |
| Yeast | P38789 | Y | Y | nucl | 33.5 | High |
| Yeast | Q06679 | Y | Y | nucl | 22.5 | High |
| Yeast | Q12024 | Y | N | nucl | 28 | High |
| Yeast | Q04031 | Y | Y | nucl | 35 | High |
| Yeast | P32529 | N | N | cyto | 10.5 | Medium |
| Yeast | P38805 | Y | Y | nucl | 32 | High |
| Yeast | P10964 | Y | Y | nucl | 26 | High |
| Yeast | P38961 | Y | Y | nucl | 30 | High |
| Yeast | Q05636 | N | N | cyto | 9 | Low |
| Yeast | P40422 | N | N | nucl | 15.5 | High |
| Yeast | P0CX38 | Y | Y | cyto | 25.5 | High |
| Yeast | Q02892 | Y | Y | nucl | 27.5 | High |
| Yeast | P39516 | Y | N | cyto | 15 | High |
| Yeast | P39744 | Y | Y | nucl | 34.5 | High |
| Yeast | P46948 | N | N | nucl | 15.5 | High |
| Yeast | Q07844 | Y | Y | nucl | 26.5 | High |
| Yeast | P53335 | Y | Y | nucl | 31.5 | High |
| Yeast | P48164 | N | Y | nucl | 21 | High |
| Yeast | Q12277 | N | N | nucl | 15.5 | High |
| Yeast | Q12481 | Y | Y | nucl | 35.5 | High |
| Yeast | P53254 | N | N | nucl | 20 | High |
| Yeast | P53972 | N | Y | nucl | 22 | High |
| Yeast | P53261 | Y | Y | nucl | 35.5 | High |
| Yeast | P53914 | Y | Y | nucl | 30 | High |
| Yeast | Q99207 | Y | Y | nucl | 39.5 | High |
| Yeast | P25382 | N | Y | nucl | 18.5 | High |
| Yeast | P40362 | N | N | nucl | 19 | High |
| Yeast | P36144 | N | N | nucl | 21.5 | High |
| Yeast | Q06511 | Y | Y | nucl | 34.5 | High |
| Yeast | P25635 | N | N | nucl | 10.5 | Medium |
| Yeast | P36160 | Y | Y | nucl | 27.5 | High |
| Yeast | Q12153 | Y | Y | nucl | 33.5 | High |
| Yeast | Q06287 | N | N | cyto | 14.333 | Medium |
| Yeast | P53883 | Y | Y | nucl | 38.5 | High |
| Yeast | Q07896 | Y | Y | nucl | 36.5 | High |
| Yeast | P32495 | Y | Y | cyto | 21.833 | High |
| Yeast | P48234 | Y | Y | nucl | 43 | High |
| Yeast | P38112 | Y | Y | nucl | 33.5 | High |
| Yeast | Q6Q547 | N | N | nucl | 20.5 | High |
| Yeast | Q06512 | N | N | cyto | 10.5 | Medium |
| Yeast | P43586 | Y | Y | nucl | 36.5 | High |
| Yeast | P38205 | Y | Y | nucl | 27.5 | High |
| Yeast | Q12460 | Y | Y | cyto | 20.833 | High |
| Yeast | Q02354 | N | Y | nucl | 20 | High |
| Yeast | Q99216 | N | N | others | 8.5 | Low |
| Yeast | P53313 | Y | Y | others | 25.666 | High |
| Yeast | Q06506 | Y | Y | nucl | 36 | High |
| Yeast | P53256 | N | N | cyto | 13.5 | Medium |
| Yeast | P28000 | N | N | nucl | 16.5 | High |
| Yeast | P22139 | N | N | nucl | 11.5 | Medium |
| Yeast | Q12754 | Y | Y | nucl | 32 | High |
| Yeast | Q06078 | Y | Y | others | 13 | Medium |
| Yeast | P53336 | N | Y | nucl | 19.5 | High |
| Yeast | Q05946 | N | N | cyto | 7 | Low |
| Yeast | P07703 | N | N | cyto | 14.5 | Medium |
| Yeast | Q08162 | N | N | nucl | 13.5 | Medium |
| Yeast | P22138 | N | N | nucl | 12.5 | Medium |
| Yeast | Q06132 | Y | Y | nucl | 27.5 | High |
| Yeast | P36080 | Y | Y | nucl | 39 | High |
| Yeast | P25582 | Y | Y | nucl | 39.5 | High |
| Yeast | Q08285 | N | N | cyto | 16 | High |
| Yeast | P46669 | N | Y | nucl | 26 | High |
| Yeast | P05755 | N | N | nucl | 14 | Medium |
| Yeast | Q12339 | Y | Y | nucl | 26 | High |
| Yeast | P40055 | N | Y | nucl | 25.5 | High |
| Yeast | Q04305 | Y | Y | others | 16 | High |
| Yeast | P40498 | Y | Y | nucl | 35 | High |
| Yeast | Q3E705 | Y | Y | nucl | 35.5 | High |
| Yeast | P15646 | Y | N | nucl | 16 | High |
| Yeast | Q03973 | Y | Y | nucl | 29 | High |
| Yeast | Q06218 | Y | Y | nucl | 27.5 | High |
| Yeast | P46962 | N | Y | others | 13 | Medium |
| Yeast | P36049 | Y | Y | nucl | 41.5 | High |
| Yeast | Q12099 | N | Y | nucl | 15 | High |
| Yeast | P29295 | N | N | nucl | 14.5 | Medium |
| Yeast | P53188 | Y | Y | nucl | 29.5 | High |
| Yeast | Q06344 | Y | Y | nucl | 40 | High |
| Yeast | P36120 | Y | Y | nucl | 37 | High |
| Yeast | Q03532 | Y | N | nucl | 24.5 | High |
| Yeast | P41819 | N | N | others | 11 | Medium |
| Yeast | P38719 | Y | Y | cyto | 19.5 | High |
| Yeast | Q06631 | N | N | nucl | 20.5 | High |
| Yeast | P40546 | Y | N | nucl | 29 | High |
| Yeast | P53743 | Y | N | nucl | 27.5 | High |
| Yeast | P40493 | Y | Y | others | 18 | High |
| Yeast | Q08965 | Y | Y | nucl | 41.5 | High |
| Yeast | Q04217 | Y | Y | nucl | 41 | High |
| Yeast | P39985 | Y | Y | nucl | 28 | High |
| Yeast | P53859 | N | N | cyto | 14.833 | Medium |
| Yeast | P38278 | N | Y | nucl | 18.5 | High |
| Yeast | Q08235 | N | Y | nucl | 21.5 | High |
| Yeast | P33750 | Y | Y | nucl | 40 | High |
| Yeast | Q12457 | Y | Y | nucl | 37.5 | High |
| Yeast | P53941 | N | N | nucl | 12.5 | Medium |
| Yeast | P33201 | N | N | nucl | 16 | High |
| Yeast | Q08208 | Y | Y | nucl | 40 | High |
| Yeast | P53742 | Y | Y | nucl | 29.5 | High |
| Yeast | P47077 | Y | Y | nucl | 28.5 | High |
| Yeast | Q12080 | Y | Y | nucl | 40.5 | High |
| Yeast | P32899 | N | N | nucl | 23 | High |
| Yeast | P20484 | Y | Y | nucl | 22.5 | High |
| Yeast | Q06214 | Y | Y | nucl | 24.5 | High |
| Yeast | P53734 | N | N | nucl | 20.5 | High |
| Yeast | P47083 | Y | Y | nucl | 32.5 | High |
| Yeast | Q12499 | Y | Y | nucl | 20.5 | High |
| Yeast | P53927 | Y | Y | nucl | 34.5 | High |
| Yeast | P40007 | Y | Y | nucl | 34 | High |
| Yeast | Q07845 | Y | Y | nucl | 23.5 | High |
| Yeast | O13329 | Y | Y | nucl | 34.5 | High |
| Yeast | P10080 | Y | N | nucl | 21.5 | High |
| Yeast | P25368 | Y | N | nucl | 26.5 | High |
| Yeast | P27476 | Y | Y | nucl | 31.5 | High |
| Yeast | P28007 | Y | Y | others | 15 | High |

^*^NoD (A), NLStradamus (B), and WoLF PSORT (C). Predictions from NoD and NLStradamus were marked “Y” for positive and “N” for negative. WoLF PSORT predictions were interpreted as follows: “nucl” for nuclear, “cyto” for cytosol, and “others” for subcellular locations other than nucleus and cytoplasm.

**Supplementary Table S4**. Examples of proteins associated with nucleolar ribosome biogenesis and rRNA modification complexes identified in *Magnaporthe oryzae* using FuNGI.

| **Complex** | **Protein name** | **Uniprot Accession** | **Predicted NoLS Segments** | **Predicted NLS Segments** | **Confidence** |
| --- | --- | --- | --- | --- | --- |
| PeBoW-associated ribosome biogenesis | MAK21 | G4N7W3 | 4 | 4 | High |
| PeBoW-associated ribosome biogenesis | BRX1 | G4NCH9 | 2 | 1 | Medium |
| PeBoW-associated ribosome biogenesis | SSF1 | G4NEI7 | 3 | 1 | High |
| PeBoW-associated ribosome biogenesis | NOP53 | G4NIR7 | 4 | 4 | High |
| PeBoW-associated ribosome biogenesis | NSA2 homolog | G4MYW1 | 2 | 2 | High |
| PeBoW-associated ribosome biogenesis | KRI1 | G4N8I5 | 5 | 5 | High |
| rRNA methylation / box C/D-related | SPB1 rRNA methyltransferase | Q52C47 | 5 | 5 | High |
| Ribosome assembly WD-repeat proteins | WD repeat protein | G4N6X7 | 2 | 2 | High |
| Ribosome assembly WD-repeat proteins | WD repeat protein 44 | G4NBW6 | 2 | 1 | Medium |

**Supplementary Table S5.** Classification performance of four machine learning algorithms (Random Forest (RF), Gradient Boosting (XGBoost), Support Vector Machines (SVM), and Logistic Regression (LR)) trained on ESM-C embeddings to distinguish positive (HumanNoLS+ and MoNoLS+) from negative (MoNoLS–) datasets.

| Model | Accuracy ^*^ | F1 ^*^ | Precision ^*^ | Sensitivity ^*^ | AUC ^*^ |
| --- | --- | --- | --- | --- | --- |
| RF | 0.93 ± 0.05 | 0.93 ± 0.05 | 0.95 ± 0.06 | 0.92 ± 0.08 | 0.97 ± 0.04 |
| XGBoost | 0.93 ± 0.05 | 0.92 ± 0.05 | 0.94 ± 0.06 | 0.92 ± 0.08 | 0.97 ± 0.03 |
| LR | 0.94 ± 0.05 | 0.93 ± 0.05 | 0.97 ± 0.04 | 0.90 ± 0.08 | 0.98 ± 0.02 |
| SVM | 0.93 ± 0.05 | 0.92 ± 0.05 | 0.96 ± 0.05 | 0.90 ± 0.08 | 0.98 ± 0.02 |

^*^Data are represented as mean ± standard deviation over 1,000 iterations of stratified 5-fold cross-validation.
